# In Vivo Metabolic Analysis of the Anticancer Effects of Plasma-Activated Saline in Three Tumor Animal Models

**DOI:** 10.3390/biomedicines10030528

**Published:** 2022-02-23

**Authors:** Miao Qi, Dehui Xu, Shuai Wang, Bing Li, Sansan Peng, Qiaosong Li, Hao Zhang, Runze Fan, Hailan Chen, Michael G. Kong

**Affiliations:** 1 State Key Laboratory of Electrical Insulation and Power Equipment, Centre for Plasma Biomedicine, Xi’an Jiaotong University, Xi’an 710049, China; qimiao@stu.xjtu.edu.cn (M.Q.); peng33@stu.xjtu.edu.cn (S.P.); liqiaosong1010@stu.xjtu.edu.cn (Q.L.); zhang216@mail.xjtu.edu.cn (H.Z.); fanrunze@stu.xjtu.edu.cn (R.F.); 2 The School of Life Science and Technology, Xi’an Jiaotong University, Xi’an 710049, China; shuaiwang3059@stu.xjtu.edu.cn (S.W.); lb905533242@stu.xjtu.edu.cn (B.L.); 3 Frank Reidy Center for Bioelectrics, Old Dominion University, Norfolk, VA 23508, USA; h1chen@odu.edu; 4 Department of Electrical and Computer Engineering, Old Dominion University, Norfolk, VA 23529, USA

**Keywords:** plasma, CAP, PAS, cancer treatment, metabolic pathway

## Abstract

In recent years, the emerging technology of cold atmospheric pressure plasma (CAP) has grown rapidly along with the many medical applications of cold plasma (e.g., cancer, skin disease, tissue repair, etc.). Plasma-activated liquids (e.g., culture media, water, or normal saline, previously exposed to plasma) are being studied as cancer treatments, and due to their advantages, many researchers prefer plasma-activated liquids as an alternative to CAP in the treatment of cancer. In this study, we showed that plasma-activated-saline (PAS) treatment significantly inhibited tumor growth, as compared with saline, in melanoma, and a low-pH environment had little effect on tumor growth in vivo. In addition, based on an ultra-high-performance liquid tandem chromatography-quadrupole time-of-flight mass spectrometry (UHPLC-QTOF-MS) analysis of tumor cell metabolism, the glycerophospholipid metabolic pathway was the most susceptible metabolic pathway to PAS treatment in melanoma in vitro and in vivo. Furthermore, PAS also inhibited cell proliferation in vivo in oral tongue squamous-cell cancer and non-small-cell lung cancer. There were few toxic side effects in the three animal models, and the treatment was deemed safe to use. In the future, plasma-activated liquids may serve as a potential therapeutic approach in the treatment of cancer.

## 1. Introduction

Melanoma is one of the most common malignant tumors in clinical practice. Although melanoma constitutes approximately 5% of all skin cancers, it accounts for more than 75% of deaths from skin cancer. With the emergence of molecular-targeted therapy and immunotherapy, the treatment of metastatic melanoma has undergone tremendous changes in the past decade [1]. Immunotherapy based on the programmed death 1 (PD-1) pathway has been effective for advanced melanoma, although the overall objective response rate has been around 33% in recent clinical studies [2], suggesting that a majority of melanoma patients may need alternative treatment strategies. Among the many approaches investigated, cold atmospheric plasma (CAP) has elicited anticancer effects in a diversity of human malignant cells and in tumors in xenograft models [3,4].

CAP has been increasingly used in novel therapies for the treatment of cancer, wounds, and other disorders over the past two decades [5] by producing reactive oxygen species (ROS), reactive nitrogen species (RNS), electrons and ions, photons, and a transient electric field [6,7]. Recently, CAP in flowing argon was found to be efficacious in reducing head and neck tumors in human patients and improving their quality of life in a pilot study via open-label clinical trials [8]. These preclinical and clinical advances highlight the potential of a novel engineering-based anticancer therapy. Interestingly, liquid pretreated by CAP, often known as a plasma-activated solution (PAS), possesses similar anticancer properties to CAP, thus facilitating its utility for treating deep-seated tumors or tumors in body cavities and significantly broadening the translation reach of CAP technology [9]. The anticancer properties of PAS have been shown in multiple tumors, including cervical cancer, pancreatic cancer, gastric cancer, colon cancer, melanoma, and triple-negative breast cancer [10,11,12,13,14,15,16,17,18,19]. PAS prepared in saline, RPMI-1640 (Roswell Park Memorial Institute-1640), and DMEM (Dulbecco’s Modified Eagle’s Medium) medium has been of particular interest, and saline and Ringer’s lactate have been approved by regulatory authorities for use in humans [20].

ROS and RNS have been major effectors of the anticancer activity of CAP and are involved in CAP-mediated apoptosis, immunogenic cell death, and cancer cell death pathways [21]. However, the current understanding of CAP-mediated anticancer mechanisms is limited, and this is even more challenging with PAS, in which anticancer activity was only recently reported. We reasoned that ROS and RNS may be major effectors in PAS, as has been reported in CAP, and that the acidity of PAS may be a complementary effector [9], which has not been seen in CAP. ROS are major players in tumor metabolism [22], whereas acidity disrupts the pH regulation of cell functions and energetics [23]. Tumor metabolism is a known hallmark of cancer [24] but has not been explored for CAP or PAS. Given these observations, we hypothesized that PAS could effectively disrupt the energy metabolism of tumors and suppress their survival. To test this hypothesis, we considered melanoma as a case study by assessing PAS efficacy in reducing melanoma in a xenograft model and PAS disruption of tumor cell metabolism by means of UHPLC-QTOF-MS analysis. Furthermore, we extended the case study of melanoma to test whether PAS, with the same chemistry, was similarly efficacious in reducing tumors of lung and oral cancer cells in xenograft models.

In this study, we found that plasma-activated saline (PAS) treatment significantly suppressed tumor growth, as compared to saline treatment on melanomas (A375 cell line) in vivo. In addition, through UHPLC-QTOF-MS analysis of tumor cell metabolism, we showed that PAS treatment was able to significantly alter the metabolite profiling of tumor cells. Notably, the glycerophospholipid metabolic pathway was the most susceptible metabolic pathway to PAS treatment in animal models. To explore whether PAS was spectral in the treatment of tumors, we conducted a verification using oral tongue squamous-cell cancer (Tca-8113 cell line) and non-small-cell lung cancer (A549 cell line) and found that PAS also had a significant effect on the treatment of these two cancers.

## 2. Materials and Methods

### 2.1. Experimental Device

**Figure 1:**
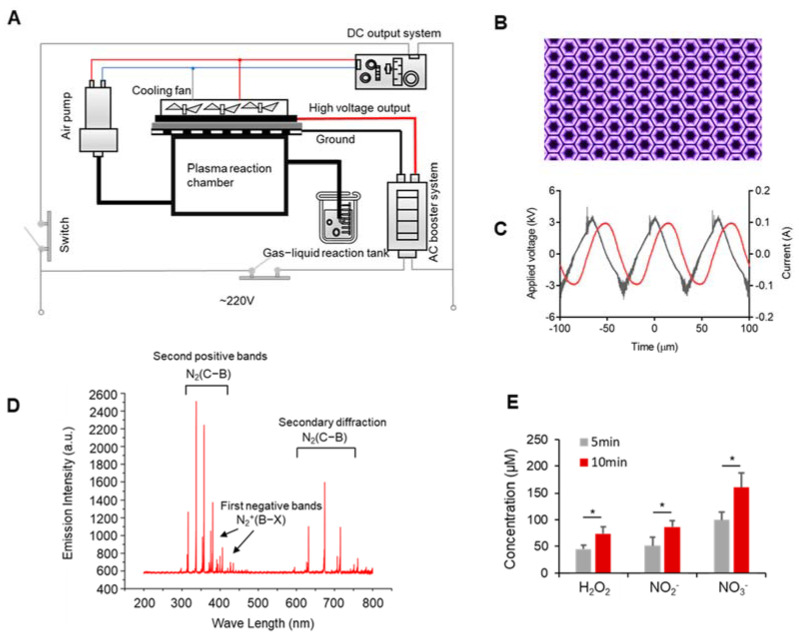
Development of the portable plasma device and detection of the discharge characteristics. (**A**) Schematic diagram of the DBD plasma device. (**B**) Plasma discharge photo. (**C**) Waveforms of the applied voltage and discharge current. (**D**) Plasma spectral emission intensity of the DBD plasma device. (**E**) The concentration of long-lived particles. Data represent the mean ± SD; *n* = 3; * *p* < 0.05.

A portable and miniaturized DBD (dielectric-barrier-discharge) plasma device was developed at our laboratory [25] with a DC (Direct Current) input, an AC (Alternating Current) booster unit, an air pump, and a cooling system (Figure 1A). See the plasma discharge photo in Figure 1B. We measured the voltage and current of the AC boost system using a high-voltage probe (Tektronix, P6015A, Beaverton, OR, USA) and a current probe (Tektronix, P6021, Beaverton, OR, USA), and observed it using an oscilloscope (Agilent, DSO-X 2014A, Santa Clara, CA, USA). The results are shown in Figure 1C, where the voltage applied to the electrodes was sinusoidal at 15 kHz with a peak-to-peak voltage of 6 kV; the total power was 40 w. Effluent from the DBD was allowed to react in a downstream reaction chamber and then left to activate saline in a container at a flow rate of 15 L/min (Figure 1A). The activation time was 10 min for 5 mL of saline for all experiments.

### 2.2. Cell Culture

A375, Tca-8113, and A549 cell lines were obtained from the American-Type Culture Collection (ATCC) (Manassas, VA, USA). A549 and Tca-8113 cell lines were cultured in RPMI-1640 (Corning, Jiangsu, China), and the A375 cell line was cultured in DMEM (Corning, Jiangsu, China) and supplemented with 10% fetal bovine serum (Gibco, NY, USA) and 1% penicillin–streptomycin (100×) (MedChemExpress, Princeton, NJ, USA) at 37 °C in a 5% CO_2_ incubator.

### 2.3. Cell Viability Assay

A total of 5000 cells of A375 were seeded in 96-well plates supplemented with complete medium. A cell-counting kit (CCK, 7sea biotech, Shanghai, China) was used to detect cell viability. After 1 day, the medium was changed so that it did not have serum overnight. 1-Oleoyl-sn-glycero-3-phosphate (LPA, C18:1, Sigma-Aldrich, Shanghai, China) was diluted in basic medium containing 0.1% fatty-acid-free bovine serum albumin (BSA, Solarbio, Beijing, China). On the next day, the cells were stimulated for 24 h with LPA (1 μM) and PAS. CCK reagent was added to each well, and the cells were incubated at 37 °C for 2 h, and then the absorbance at 570 nm was recorded using a microplate reader (Varioskan Flash, Thermo, Waltham, MA, USA). Independent experiments were repeated in triplicate.

### 2.4. Animal Study

**Figure 2:**
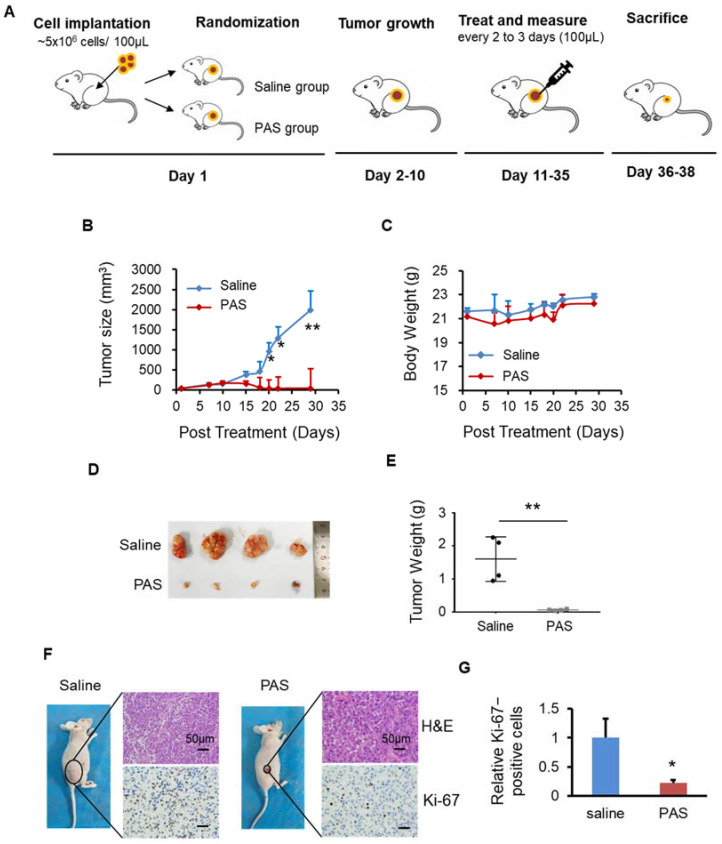
Plasma-activated saline (PAS) injection significantly suppressed A375 xenograft tumor growth in vivo. (**A**) Illustration of the three tumor models. Nude mice bearing A375 xenografts were grouped and treated with saline or PAS. The tumor size (**B**) and body weight (**C**) were measured. After the experiment was terminated, tumors were harvested (**D**) and weighed (**E**). Tumor sections were stained with hematoxylin and eosin (H&E) (upper panel), Ki-67 (lower panel) (**F**). (**G**) Quantitative analyses of Ki67-positive cells were performed with ImageJ. Data represent the mean ± SD; *n* = 4; * *p* < 0.05; ** *p* < 0.01; scale bar = 50 μm.

BALB/c nude mice (female), 6–8 weeks old, were purchased from the Medical Animal Center of Xi’an Jiaotong University. Mice were allowed to adapt for one week prior to the experiment. Mice were randomly divided into two groups (saline and PAS) or three groups (saline, PAS, and saline + HCl) groups, with four mice per group. In total, 100 μL of cells (5 × 10^6^) were subcutaneously implanted into the right flank of nude mice. Tumor length (L) and width (W) were measured three times per week to determine the tumor volume using the formula (L × W^2^)/2 and presented as mean ± SD. Tumors were allowed to grow to 50 mm^3^ and were then subcutaneously treated 3 times per week with 100 μL of saline or PAS (after 5 mL of saline were activated for 10 min, 100 μL of PAS were immediately injected into the tumor of the mice, within 3 min from the preparation to the end of use). As the time for the tumors of the 3 different cancer cells to reach 50 mm^3^ differed from 1 group to another, the endpoint for each cancer cell line was different. At the specific endpoint of each cell line, the mice were euthanized, and tumors were harvested for weight, and immunohistochemistry analysis was conducted. The experimental protocols were approved by the Hospital Research Ethics Committee of Xi’an Jiaotong University. Figure 2A shows an illustration of the three tumor models.

### 2.5. Immunohistochemical Staining

After weighing, the tumors were fixed in 10% neutral-buffered formalin overnight and embedded in paraffin (FFPE). Sections of 5 µm thickness were cut, dried in a 60 °C oven overnight, deparaffinized, rehydrated, and stained with hematoxylin, eosin, and Ki-67 antibody. The sample was heated twice in a 10 mM sodium citrate aqueous solution in a microwave oven for 20 min. Immersing the sections in 5% hydrogen peroxide methanol (3 × 10 min) blocked endogenous peroxidase activity. Then, the sections were washed twice with 1× phosphate-buffered saline (PBS, pH 7.4) and incubated in 1% BSA. The primary antibody for Ki-67 (Proteintech, Wuhan, China) was incubated at 4 °C overnight. At room temperature, washing with PBS and incubation with secondary antibody for 30 min were carried out. The final staining was completed in diaminobenzidine tetrahydrochloride (DAB) solution to visualize positive staining, and hematoxylin was used for counterstaining. Sections were imaged with an OLYMPUS BX53 microscope. We used 5 graphs from each group to analyze relative ki-67-positive cells using ImageJ software.

### 2.6. UHPLC-QTOF-MS Analysis

A total of 50 mg of sample were weighted to an EP tube, and 1000 μL of extract solution (acetonitrile/methanol/water = 2:2:1, with isotopically labeled internal standard mixture) were added. After 30 s of vortexing, the samples were homogenized at 35 Hz for 4 min and sonicated for 5 min on ice. The homogenization and sonication cycles were repeated 3 times. Then, the samples were incubated for 1 h at −40 °C and centrifuged at 12,000 rpm for 15 min at 4 °C. The resulting supernatant was transferred to a fresh glass vial for UHPLC-QTOF-MS analysis.

This experiment used a 1290 Infinity series UHPLC System (Agilent Technologies, CA, USA) equipped with a UPLC BEH Amide column (2.1 × 100 mm, 1.7 μm, Waters). The mobile phase consisted of phase A: containing 25 mmol/L ammonium acetate and 25 mmol/L ammonia hydroxide in water (pH = 9.75) and phase B was acetonitrile. We used the following gradient elution: 0–0.5 min, 95% B; 0.5–7 min, 95%–65% B; 7–8 min, 65%–40% B; 8–9 min, 40% B; 9–9.1 min, 40%–95% B; and 9.1–12 min, 95% acetonitrile. The mobile phase flow rate was 0.5 mL/min with a column temperature of 25 °C, sample tray temperature of 4 °C, and injection volume of 3 μL.

The Thermo Q Exactive Orbitrap mass spectrometer can collect primary and secondary mass spectrometry data using control software (Xcalibur, version: 4.0.27, Thermo). The detailed parameters were as follows: sheath gas flow rate, 45 Arb; Aux gas flow rate 15 Arb; capillary temperature, 400 °C; full MS resolution, 70,000; MS/MS resolution, 17,500; collision energy, 10/30/60 in NCE mode; and spray voltage, 4.0 (positive) or −3.6 kV (negative).

### 2.7. Emission Spectrum Detection

A UV-Vis spectrometer (Maya pro2000, Ocean Optics, Shanghai, China) was used to measure the surface discharge emission spectra. The detection wavelength range was 200–800 nm. We placed the spectrometer probe approximately 2 cm away from the surface discharge area to ensure clarity and accuracy of the spectrum detection. 

### 2.8. Statistical Analysis

Metabolome original data were converted into mzXML format by ProteoWizard software with an in-house program, which was developed using R and based on XCMS, for peak identification, extraction, and integration, and then an s an in-house MS2 database (BiotreeDB) was applied in metabolite annotation. The cutoff for annotation was set at 0.3. Other results are presented as the means ± SD. To determine the significance between the tested groups, the Student’s t-test was used. Data from studies were considered statistically significant different at * *p* < 0.05 and ** *p* < 0.01.

## 3. Results

### 3.1. Discharge Plasma and Aqueous Reactive Species Generation

Using a 220 V AC voltage as the power input, the absorption spectra of the plasma plumes of a DBD plasma device were detected (Figure 1D). Figure 1B shows that the surface discharge plasma has many radiation lines and an emission spectrum from 200 to 800 nm. The spectrum was mainly composed of the first negative bands of N_2_^+^(B^2^Σu^+^→X^2^Σ_g_^+^), the second positive bands of N_2_(C^3^∏_u_→B^3^∏_g_), and the secondary diffraction of N_2_(C^3^∏_u_ →B^3^∏_g_). The specific data of the spectral emission intensity are shown in Table S1. The plasma contained a wide variety of reactive particles. After reaching the liquid surface, these meteorologically reactive particles generated a variety of liquid-phase ROS through gas–liquid and liquid–liquid interactions, such as long-lived particles (H_2_O_2_, NO_2_^−^, and NO_3_^−^) and short-lived particles (OH•, O_2_^−^, and ONOO^−^). As most of the gas exiting the tube consisted of long-lived particles, we detected the concentration of long-lived particles. As shown in Figure 1C, the H_2_O_2_, NO_2_^−^, and NO_3_^−^ concentrations in normal saline increased from 44.8 to 37.65 µM, from 51.2 to 86.67 µM, and from 99.76 to 160.8 µM, respectively. Therefore, the concentrations of long-lived particles increased with the plasma-discharge time.

### 3.2. PAS Inhibited Melanoma Cell Growth In Vivo

To prove the effects of PAS on melanoma, as shown in Figure 2A, after 10 days of PAS treatment in mice, the A375 tumor volume began to decline. The growth of PAS-treated tumors was significantly suppressed by approximately 98% (Figure 2B). The body weight of the mice in the saline and PAS groups showed no significant changes (Figure 2C), suggesting few toxic side effects from the PAS injection. The volume and weight of the saline group tumors were significantly higher than the PAS group tumors (Figure 2D); specifically, the average weight of the saline group tumors was 1.65 g and that of the PAS tumors was 0.06 g. The tumor weight for the PAS group was around 4% of the tumor weight for the saline-injected group (Figure 2E). Immunohistochemical (IHC) staining showed that the positive rate of Ki-67 in tumor cells of the PAS group was significantly lower (Figure 2F,G). Our data indicated that PAS showed significant antitumor efficacy in human melanomas in vivo.

### 3.3. PAS Inhibited Cancer Cell Growth In Vivo in a pH-Independent Manner

**Figure 3:**
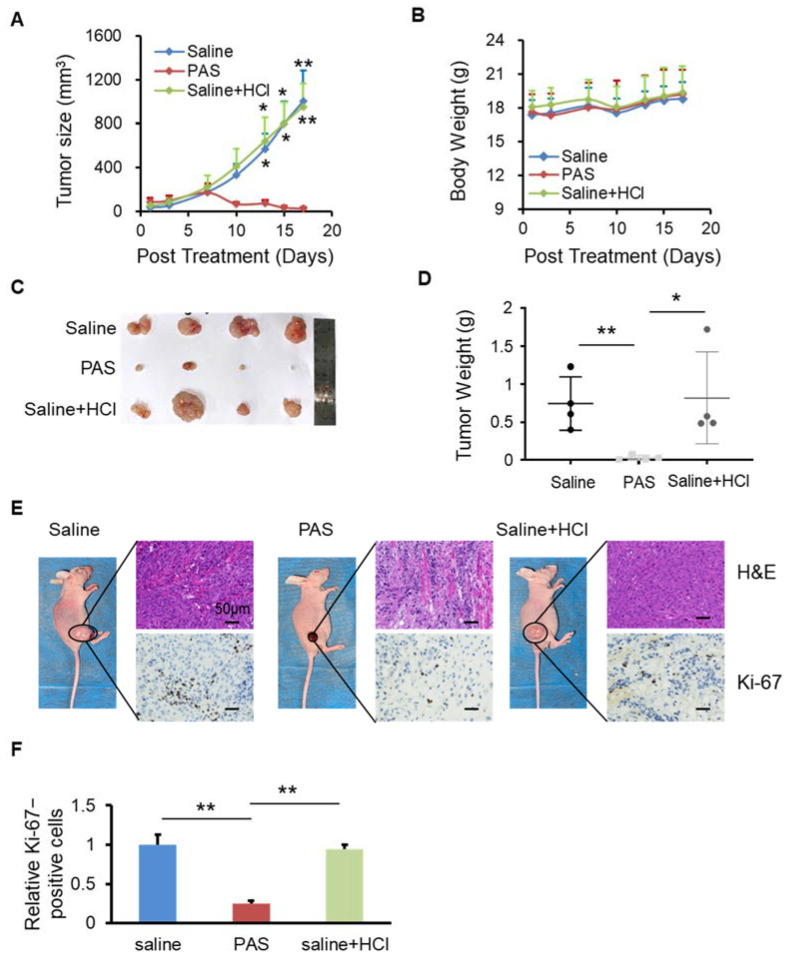
PAS injection suppressed cell growth that was independent of the pH value. Nude mice bearing A375 xenografts were grouped and treated with saline, PAS, or HCl used to adjust saline pH to 2.5. The tumor size (**A**) and body weight (**B**) were measured. After euthanasia, tumors were harvested (**C**) and weighed (**D**). Tumor sections stained with H&E (upper panel) and Ki-67 (lower panel) (**E**). (**F**) Quantitative analyses of Ki67-positive cells were performed with ImageJ. Data represent the mean ± SD; *n* = 4; * *p* < 0.05; ** *p* < 0.01; scale bar = 50 μm.

After the saline was activated by plasma for 10 min, there were many reactive particles, such as H^+^, NO_2_^−^, NO_3_^−^, OH^−^, etc., that caused the pH of the PAS to drop to approximately 2.5. To verify whether the inhibitory effect of PAS on tumors was related to the pH of the saline, we used concentrated hydrochloric acid to adjust the pH of the brine to 2.5, as shown in Figure 3A. At 28 days after the saline, PAS, or saline + HCl injections, the PAS treatment significantly suppressed melanoma growth: the volume of the tumors decreased from 51 to 20 cm^3^, which was a dramatic inhibition of approximately 60%. However, the saline + HCl treatment (53–953 cm^3^) displayed no significant changes, as compared to the saline group (46–1001 cm^3^). During treatment, there was almost no difference in the body weights or side effects between the saline, PAS, and saline + HCl groups (Figure 3B). After 28 days of treatment, the relative volume and weight of the tumors from the PAS treatment were dramatically lower than the saline treatment, and the tumors that received the saline + HCl treatment were basically unchanged, as compared to those of the saline group (Figure 3C). On average, the weight of the tumors from the PAS treatment mice was 0.03 g, a decrease of 96% as compared to the saline group. The average weight of the tumors from the saline + HCl treatment mice was 0.81 g, and that of the saline mice was 0.75 g. There was no significant difference between the saline + HCl and saline groups (Figure 3D). IHC staining showed that the positive rate of Ki-67 in the tumor cells of the PAS treatment group was significantly lower, and those of the saline and saline + HCl groups were basically the same (Figure 3E,F). Altogether, these data indicated that PAS inhibited cancer cell growth and proliferation in vivo in a pH-independent manner.

### 3.4. The Glycerophospholipid Metabolic Pathway Was the Most Susceptible Metabolic Pathway of PAS Therapy In Vivo

**Figure 4:**
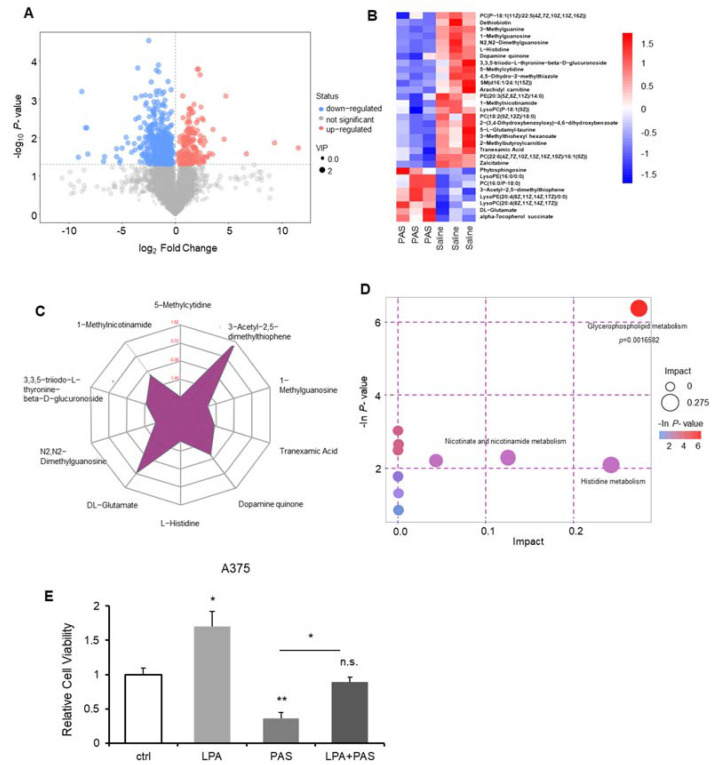
PAS regulated tumor growth in vivo through the glycerophospholipid metabolic pathway. Nude mice bearing A375 xenografts were treated with saline and PAS. (**A**) Volcano plot of the differential metabolite screening. (**B**) Cluster analysis of all differential metabolites. (**C**) Radar chart analysis of PAS vs. saline groups. (**D**) Metabolic pathway related to differential metabolites. (E) Cell proliferation was assessed using a cell counting kit (CCK) assay after the cells were treated with LPA (1 μM) and PAS for 24 h. Data represent the mean ± SD; *n* = 3; * *p* < 0.05; ** *p* < 0.01.

Tumorigenesis depends on the direct and indirect consequences of carcinogenic mutations, that is, the reprogramming of cell metabolism [24]. Altered metabolism in cancer cells is pivotal for tumor growth. To understand the PAS treatment’s inhibition of cancer proliferation, we used ultra-high-performance liquid tandem chromatography-quadrupole time-of-flight mass spectrometry (UHPLC-QTOF-MS) to investigate the differences between metabolic levels when the tumors were treated with saline and PAS. Represented by melanomas, we harvested the three tumor tissues from the saline and PAS groups, as shown in Figure 4A. Each point on the volcano map represents a metabolite. Red represents significantly upregulated metabolites, blue represents significantly downregulated metabolites, and gray represents non-significantly different metabolites. These data suggest that after PAS treatment, the metabolites had significantly changed. We screened all the upregulated and downregulated differential metabolites and used hierarchical clustering analysis to classify the metabolites with similar and different characteristics. After comparing the saline and PAS treatments, the results were visualized in a heat map (Figure 4B). There were significant changes in 31 metabolites after the PAS treatment. Taking advantage of radar-chart analysis using the heat-map mean values (Figure 4C), we found that 3-Acetyl-2,5-dimethylthiophene and DL-Glutamate were the most obviously upregulated, and 5-Methylcytidine, N2, N2-Dimethylguanosine, L-Histidine, and 1-Methylguanosine were the most obviously downregulated metabolites. Complex metabolic reactions and their regulation in organisms do not occur in isolation. Complex pathways and networks are often formed by different genes and proteins, and their mutual influence and mutual regulation ultimately lead to systematic changes in the metabolome. We examined all the pathways involved in differential metabolites that had been identified based on KEGG annotation analysis. We further screened the pathways to discover the 10 key pathways that were the most relevant to the metabolite changes. As shown in Figure 4D, the glycerophospholipid metabolic pathway had the highest correlation with differential metabolites. The database of all the metabolites is listed in Tables S2–S5. Lysophosphatidic acid (LPA) is a bioactive lipid. Structured by a glycerol, a fatty acid, and a phosphate, LPA forms phosphatidic acid (PA), which in turn is required for the biosynthesis of glycerophospholipids [26]. A375 cells treated with LPA showed that LPA significantly promoted A375 cell proliferation, and when treated with PAS, A375 cell growth was significantly inhibited. In addition, we utilized the A375 cells for treatment with PAS on the basis of the presence of LPA, and the results showed that the PAS treatment attenuated LPA-induced tumor cell growth (Figure 4E).

### 3.5. Biological Safety of PAS Injection

**Figure 5:**
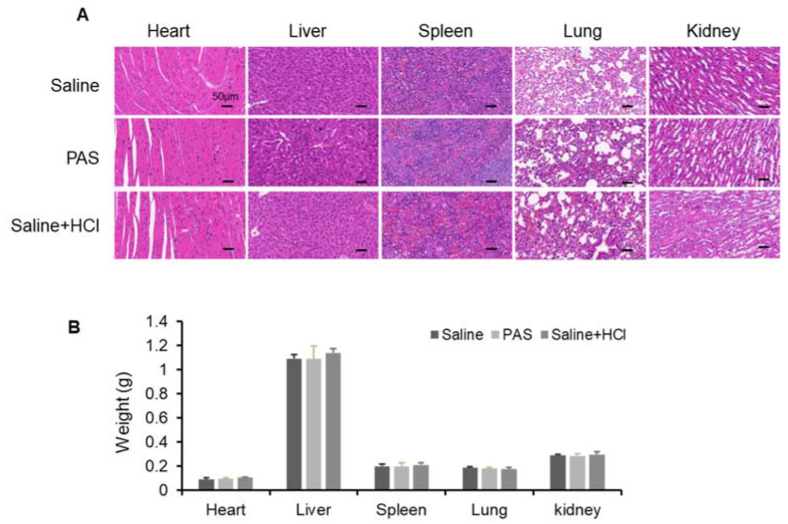
Biological safety test after long-term PAS injection. Nude mice bearing A375 xenografts were treated for 18 days with saline, PAS, or HCl used to adjust saline pH to 2.5. (**A**) The histomorphological features of murine organ slices after PAS treatment. (**B**) Effect of PAS on organ weight. Data represent the mean ± SD; *n* = 4; scale bar = 50 μm.

To evaluate the safety of PAS in vivo, several vital organs were monitored. Heart, liver, spleen, lung, and kidney tissues were harvested and stained with hematoxylin and eosin (H&E) (Figure 5A). No obvious abnormal differences were observed among the PAS, saline, and saline + HCl groups. In addition, as shown in Figure 5B, the organ weights were measured (i.e., kidneys, liver, spleen, heart, and lungs), and there was no difference regardless of the treatment. These data indicate that the PAS treatment did not affect murine organ function or weight. To further confirm the effect of PAS on vital organs, as shown in Table 1, Table 2 and Table 3, we examined blood biochemical indices of liver and kidney function, and myocardial zymograms. The results show that the saline, PAS, and saline + HCl treatments had no significant effect on the murine liver, kidney, or myocardium function, indicating that PAS treatment did not affect the blood biochemical indices of the mice. Our data demonstrated that PAS (plasma-activated normal saline for 10 min) had minimal toxic side effects and was safe to use in vivo.

Table 1, Table 2 and Table 3. Blood biochemical indices after long-term PAS injection. Nude mice bearing A375 xenografts were treated for 18 days with saline, PAS, or HCl used to adjust saline pH to 2.5. Table 1: ALT—alanine aminotransferase; AST—aspartate aminotransferase; AKP—alkaline phosphatase. Table 2: BUN—blood urea nitrogen; UA—uric acid; CR—creatinine. Table 3: LDH—lactate dehydrogenase; LDH1—lactate dehydrogenase isozyme 1; CK—creatine kinase. Data represent mean ± SD; *n* = 4.

### 3.6. PAS Suppressed Oral Tongue Squamous-Cell Carcinoma (OTSCC) Cell Growth In Vivo

**Figure 6:**
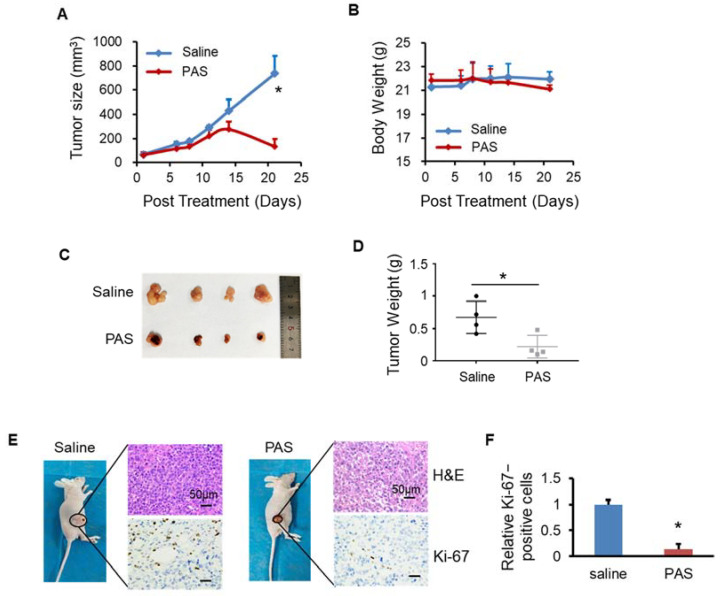
PAS injection significantly suppressed Tca-8113 xenograft tumor growth in vivo. Nude mice bearing Tca-8113 xenografts were grouped and treated with saline or PAS. The tumor size (**A**) and body weight (**B**) were measured. At the end of the experiment, tumors were harvested and weighed (**C**,**D**). Tumor sections were stained with H&E (upper panel) and Ki-67 (lower panel) (**E**). (**F**) Quantitative analyses of Ki67-positive cells were performed with ImageJ. Data represent the mean ± SD; *n* = 4; * *p* < 0.05; scale bar = 50 μm.

Oral tongue squamous-cell carcinoma (OTSCC) is the most malignant and most harmful tumor of the head and neck, and the occurrence of cases accounts for approximately 50% of head and neck squamous-cell carcinomas. As shown in Figure 6A, the PAS-injected group showed significantly inhibited Tca-8113 tumor growth (by approximately 82%) compared with the saline-injected group. To assess the toxicities mediated by PAS injection treatment in vivo, the mouse body weight was measured every 3 days and the results showed no significant differences among the various groups (Figure 6B), suggesting that the PAS injection treatment had few toxic side effects on those mice. When the experiments were terminated, the tumors were harvested and weighed. As shown in Figure 6C,D, on average, the weight of Tca-8113 tumors from the PAS-injected mice was only 33% of the tumor weight from the saline-injected mice. The PAS injection treatment significantly suppressed tumor growth compared with the saline group, further indicating that PAS shows significant antitumor efficacy in human cancer in vivo. Furthermore, IHC staining showed that the positive rate of Ki-67 in tumor cells from the PAS-injected treatment group was significantly lower (Figure 6E,F), indicating that PAS played a role in inhibiting OTSCC growth.

### 3.7. PAS Inhibited Non-Small-Cell Lung Cancer (NSCLC) Cell Growth In Vivo

**Figure 7:**
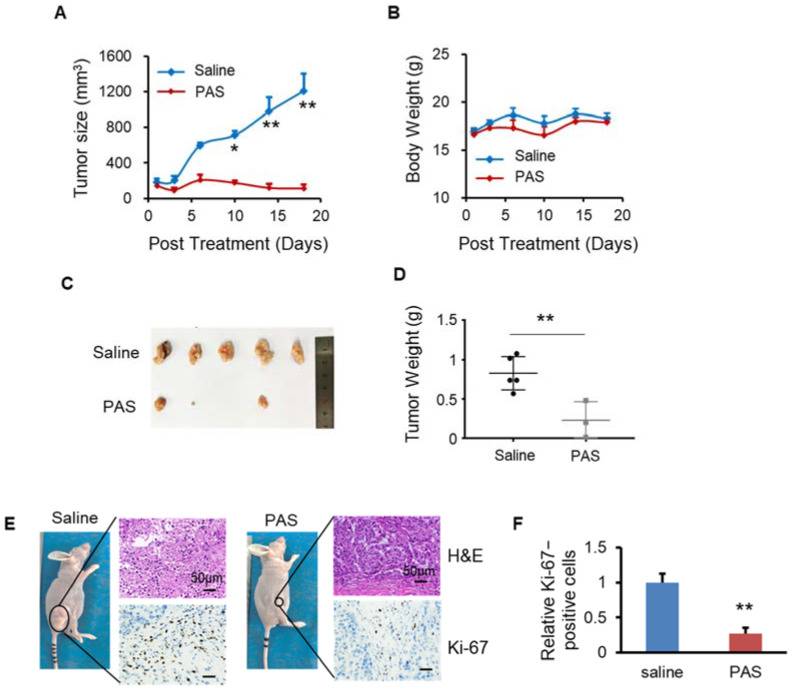
PAS injection significantly suppressed A549 xenograft tumor growth in vivo. Nude mice bearing A549 xenografts were grouped and treated with saline or PAS. The tumor size (**A**) and body weight (**B**) were measured. At the end of the experiment, tumors were harvested and weighed (**C**,**D**). Tumor sections were stained with H&E (upper panel), Ki-67 (lower panel) (**E**). (**F**) Quantitative analyses of Ki67-positive cells were performed with ImageJ. Data represent mean ± SD; *n* = 5; * *p* < 0.05; ** *p* < 0.01; scale bar = 50 μm.

Non-small-cell lung cancer (NSCLC) is the most common malignancy worldwide. It is usually diagnosed at an advanced stage and remains the leading cause of cancer-related death worldwide. In this study, we evaluated the anticancer effects of PAS on NSCLC cell lines (A549) in vivo. Regarding the antitumor effect of PAS on solid tumors, the PAS group showed significantly inhibited A549 tumor growth (by approximately 91%) compared with the saline group (Figure 7A). The mice from the two groups maintained a comparable body weight during PAS treatment (Figure 7B), suggesting few or no side effects associated with PAS treatment. In addition, the anticancer effects of the drug treatment were also confirmed by measuring the tumor weight. When the experiments were terminated, the tumors were harvested and weighed. Compared with the saline group, the PAS group showed significantly lower tumor weights (Figure 7C,D), and IHC staining showed that the positive rate of Ki-67 in tumor cells of the PAS-injected treatment group was significantly lower (Figure 7E,F), further indicating that PAS shows significant antitumor efficacy in human NSCLC in vivo.

## 4. Discussion

Among the many medical applications of cold plasma (e.g., skin disease, tissue repair, etc.), cancer is the most urgent health problem to be considered [27,28]. Increasing evidence suggests that CAP induces the death of various types of cancer cells and thus offers a promising alternative treatment. CAP has shown its potential as a selective anti-cancer tool [29]. Preliminary research in the past decade has shown that CAP can effectively inhibit the growth of dozens of cancer cells in vitro, mainly by triggering apoptosis [30]^.^

Melanoma is one of the deadliest skin cancers, which has a low incidence but an upward trend and a high degree of malignancy and poor prognosis [31]. At present, the most effective way to treat melanoma is through adjuvant chemotherapy after surgical resection, although conventional chemotherapy drugs for the clinical treatment of melanoma often have poor outcomes. Due to the toxic side effects, patients can have serious adverse reactions, and the occurrence of primary or acquired drug resistance has been reported [32]. Plasma has had a significant effect on the treatment of melanoma. Melanoma stimulates the immune system and leads to the formation of several new cancer antigens due to its high mutation rate [33]. Immune checkpoint inhibitor therapy in malignant melanoma has been reported to have anticancer effects. Gas plasma jet technology has been shown to provide immune protection against malignant melanomas both in vitro and in vivo. The treatment depth limits direct CAP irradiation. Bekeschus et al. demonstrated that vaccination with plasma-treated cells protected against melanoma growth [21]. Chen et al. studied therapies using CAP-mediated immune checkpoint blockades (ICBs) and integrated them with microneedles for the transdermal delivery of ICB [34]. Plasma-activated liquids (PALs) can be injected into or near the site of deeper tumors, can be stored for a long time, and can then be used without dependence on a CAP device. Furthermore, our findings indicated that plasma-activated saline (PAS) treatment significantly suppressed tumor growth, as compared to saline, of melanomas in vivo.

CAP can cause acidification in water and solution. The synergistic effects of induced ROS, RNS, and acidic conditions affect the intracellular Ca^2+^ level of melanoma cells, and CAP-treated solutions cause nitrification of the protein in cells under acidic conditions, which then increases the antitumor effect [35]. However, in our study, we adjusted the pH of the saline to 2.5 as a negative control, and the data shown in Figure 3 indicate that PAS inhibited cancer cell growth and proliferation in vivo in a pH-independent manner. As the microenvironment of tumors is often acidic, further acidification may have played a role in the specific anticancer effects of PAS, but acidification alone was not the main reason for its anticancer functions.

The mechanisms by which CAP effectively resists the growth of xenograft tumors implanted subcutaneously in mice are not yet fully understood. The reactive particles originating in CAP have been considered as the main factors that cause cell death. The current molecular mechanism of anticancer research has shown that the apoptosis of cancer cells caused by CAP has mainly been due to the significant increase in intracellular ROS, which in turn causes DNA double-strand breaks and a weak increase in ROS in homologous normal cells; therefore, CAP selectively causes cancer cell apoptosis in vitro [36]. Studies have shown that the differential expression of aquaporins (AQPs) and intracellular antioxidant enzymes, such as catalase, in cancer and normal cells may represent an effort to control the selective diffusion of reactive particles across the cytoplasmic membrane of cancer cells and intracellular ROS selectivity in cancer [37]. ROS are oxygen-containing molecules with high reactivity, including hydroxyl (OH•), superoxide (O_2_^−^), hydrogen peroxide (H_2_O_2_), and so on. ROS are products generated as a consequence of metabolic reactions in the mitochondria, the peroxisome, and the endoplasmic reticulum [38]. In normal cells, low-level concentrations of these compounds are required for signal transduction before their elimination. However, cancer cells, which exhibit an accelerated metabolism, demand high ROS concentrations to maintain their high proliferation rate [39]. Tumor cells harbor genetic alterations that promote continuous and elevated production of ROS. Whereas such oxidative stress conditions would be harmful to normal cells, they facilitate tumor growth in multiple ways by causing DNA damage and genomic instability, and ultimately, by reprogramming cancer cell metabolism. ROS are involved in multiple tumor metabolic pathways, including glycolysis, fatty-acid oxidation, the pentose–phosphate pathway, glutaminolysis, and the serine–glycine one-carbon metabolism [40].

Cancer metabolism is one of the oldest research fields in cancer biology. It was discovered approximately 50 years earlier than the discovery of oncogenes and tumor suppressors. Tumors reprogram pathways of nutrient acquisition and metabolism to meet the bioenergetic, biosynthetic, and redox demands of malignant cells [41]. The metabolic adaptations of cancer cells have malignant characteristics, as some altered metabolic characteristics have commonly been observed in many types of cancer cells; therefore, reprogrammed metabolism is considered to be a marker of cancer [42]. An altered metabolism is a universal property of most cancer cells [43]. For example, glucose metabolism, glutamine metabolism, and fatty-acid metabolism result from deregulated oncogenic and tumor-suppressive signal transduction pathways, and metabolic defects lead to tumor malignancy, metastasis, and drug resistance by supplying energy, building blocks, and redox potentials [44]. 

Among the various mechanisms leading to tumor cell apoptosis, metabolism is a key factor in, for example, the regulation of cell apoptosis through glycolysis, lipid metabolism, P53, and glucose metabolism [45]. Metabolism supports tumor initiation and progression through ATP, NADPH (Nicotinamide Adenine Dinucleotide Phosphate), the products of the TCA cycle, nucleotide synthesis, and electrons, which participate directly in tumor growth. Therefore, the targeting of metabolism has been used to improve cancer treatments [46]. However, the research on the metabolism of PAL in vivo remains uncertain. In this study, based on the UHPLC-QE-MS analysis of tumor cell metabolism, we observed that the glycerophospholipid and histidine metabolic pathways were the most susceptible metabolic pathways to PAS treatment in vitro and in vivo. In addition to regulating cell signaling pathways, plasma also affects the metabolism of cancer cells. Glycerophospholipids are the most abundant phospholipids and the source of physiologically active compounds, which have the highest content in all cell membranes. [47]. Transcriptomic and lipidomic analyses were used to evaluate the changes in lipid metabolism in a transgenic zebrafish model of oncogenic RAS-driven melanocyte neoplasia progression, and they showed that the glycerophospholipid pathway was deregulated in melanoma nodules. There were heterogeneous phospholipids in the tumor nodules, including an increase in phosphatidylethanolamine and phosphatidylcholine [48]. Curcuma rhizomes exhibit versatile biological activities, including significant anticancer properties. After curcuma treatment, changes were observed in the expression of 25 metabolites in A549 cells, which were involved in glycerophospholipid catabolism [49]. The high growth of cancer cells required de novo synthesis of fatty acids to continually provide glycerophospholipids, particularly for membrane production. In addition, increased lipogenesis has been recognized as a marker of cancer, and enzymes involved in de novo fatty acid synthesis and in the glycerophospholipid pathway have been used as potential targets for antitumor interventions [26].

Cold atmospheric plasma treatment could significantly alter the metabolite profiling of tumor cells, such as the beta-alanine metabolic pathway, glutaminase activity, and glucose metabolites [50,51]. Plasma produces ROS and RNS, and these function as “redox messengers” in intracellular metabolism regulation and signaling [52]. ROS causes damage to lipids, proteins, nucleic acids, and other vital components of biosystems. Shadyro et al. focused on ROS-induced reactions occurring in polar components of glycerophospholipids. When they conducted free-radical fragmentation of glycerophospholipids and the respective modeling compounds, these reactions affected cell proliferation, resistance to stress, and the functioning of mitochondria and cellular metabolisms [53]. Shadyro et al. also demonstrated that hydroxyl-containing glycerophospholipids, cardiolipin, lysolipids, and others underwent fragmentation when forming biologically active products, such as ROS, and initiated free-radical fragmentation of glycerophospholipids’ polar parts while phospholipaseA2 catalyzed the formation of lysolipids [54].

Histidine undergoes various metabolic transformation processes in the body. The most important ones are (1) decarboxylation to generate histamine, (2) the supply of methyl groups from methionine to methyl histidine, (3) the formation of imidazoline by the transamination of amino acids, and (4) the generation of glutamic acid through a series of reactions [55]. It has been reported that glutamine is an important carbon and nitrogen source in the cell and that it plays an important role in the anabolism of tumor cells. It can also be catalyzed by glutaminase (GLS) to glutamic acid and further converted to α-ketoglutarate through the tricarboxylic-acid cycle to provide energy for metabolism. Studies have shown that by inhibiting glutamate metabolism, it effectively induced tumor cell apoptosis or autophagy [56]. Kanarek et al. reported that the degradation of histidine increased the sensitivity of cancer cells to drugs and lowered the side effects of drug treatments in murine models [57].

In our study, PAS treatment significantly suppressed tumor growth, as compared to saline treatment, of melanomas in vivo. In addition, the glycerophospholipid metabolic pathway was the most susceptible metabolic pathway to PAS treatment in animal models. To explore whether PAS was spectral in the treatment of tumors, we conducted verification in oral tongue squamous-cell cancer and non-small-cell lung cancer cells and found that PAS also had a significant effect on the treatment of these two cancers. Moreover, the occurrence and development of these two cancers were inseparable from the glycerophospholipid metabolic pathway. In oral tongue squamous-cell cancer, Dickinson et al. performed metabolic analysis between the tumor tissue and healthy oral tongue mucosa samples from 10 oral squamous-cell carcinoma (OSCC) patients, where the glycerophospholipid metabolism was aberrant [58]. Preoperative plasma samples were collected from 50 patients with OSCC and 50 normal patients by lipid profiling; all glycerophospholipids were decreased [59]. In lung cancer, Hoang et al. analyzed the metabolomics of 24 fresh frozen specimens from patients with biopsy-confirmed lung adenocarcinoma. The following tissues of each patient were analyzed: tumor, near tumor, and normal lung. They found metabolites in tumor-specific characteristics, especially, as compared to normal lung, and a variety of phosphatidylethanolamines showed significant differential expression in tumor tissue [60]. Wu et al. used compound Kushen injections (CKI) to treat non-small-cell lung cancer; the tumor volume and weight of the mice in the CKI-treated group were significantly reduced. After metabolome sequencing, they found that the therapeutic effect of CKI on non-small-cell lung cancer impacted glycerophospholipid metabolism [61]. Mitchell et al. used mass spectrometry to examine differences in the lipid profiles between paired cancerous and noncancerous lung tissue samples from 86 patients with a suspected diagnosis, and they found a low abundance of the glycerophospholipid metabolic phenotype in the NSCLC samples [62].

Compared to fibroblasts, CAP kills the cells in oral squamous-cell carcinoma without damaging the fibroblasts. In addition, this effect also depends on the content of catalytic iron (II), especially in lysosomes. CAP has been shown to significantly inhibit the colony-forming ability, migration, and invasion of oral squamous-cell carcinoma cells [63]. Jae Won Chang et al. indicated that CAP induced apoptotic cell death in wild-type p53 oral squamous-cell carcinoma cells through a novel mechanism involving DNA damage and the triggering of sub-G1 arrest via the ATM/p53 pathway. These findings showed the therapeutic potential of CAP in oral squamous-cell carcinoma [64]. Han et al. developed a microwave plasma jet system where NO-plasma-activated water significantly increased cytotoxicity and apoptosis, and then the expression levels of caspase and PARP were increased in OSCC [65].

CAP and PAM can induce the apoptosis of lung cancer cells. Ma et al. showed that H_2_O_2_ was generated in the plasma-activated solution, which in turn induced apoptosis and DNA double-strand breaks in A549 cells. CAP also caused mitochondrial dysfunction and intracellular ROS in A549 cells. In addition, catalase effectively protected cells from CAP-induced A549 cell damage [66]. The synergistic effect of CAP and iron-oxide-based magnetic nanoparticles also effectively killed lung cancer cells and significantly inhibited cell proliferation by reducing viability and inducing apoptosis. The combination of the two induced EGFR (Epidermal Growth Factor Receptor) downregulation, whereas CAP inhibited lung cancer cells by inhibiting p-ERK and p-AKT [67]. Jo et al. indicated a plasma-activated medium produced by a microwave-excited atmospheric-pressure plasma jet with argon rapidly increased the intracellular ROS of A549 cells and induced apoptosis [68]. In addition, the mitochondrial–nuclear network induced an apoptotic cascade in A549 cells [69].

In future studies, we will explore other cancer types in vivo to detect the concentrations of other reactive particles and perform correlation analyses with tumor suppression to obtain the main reactive particles that are critical for tumor ablation, which may provide direction for overcoming obstacles when applying this treatment to in vivo systems and ensuring the optimal control of plasma devices.

## 5. Conclusions

In this study, we demonstrated that PAS treatment dramatically inhibited tumor growth, as compared to a control group, in melanoma and independent of the pH level. According to the weight of mice, their organs (i.e., heart, liver, spleen, lung, kidney) and H&E staining, and the blood biochemical index, including liver function, kidney function, and myocardium zymogram, the PAS treatment (plasma-activated normal saline for 10 min) had insignificant toxic side effects and was safe for use in vivo. In addition, according to a UHPLC-QTOF-MS analysis of the tumor cell metabolism, we showed that the glycerophospholipid metabolic pathway was the most susceptible metabolic pathway to PAS treatment in tumor cells. Furthermore, PAS also inhibited cell proliferation in vivo in oral tongue squamous-cell cancer and non-small-cell lung cancer.

## Figures and Tables

**Table 1:** Analysis of liver function.

	ALT (U/L)	AST (U/L)	AKP (U/L)
Saline	101.44 ± 14.86	399.35 ± 53.87	95.01 ± 13.77
PAS	101.23 ± 5.93	403.62 ± 49.71	98.78 ± 7.59
Saline + HCl	99.58 ± 15.93	395.21 ± 68.85	95.04 ± 17.58

**Table 2:** Analysis of kidney function.

	BUN (mg/dL)	UA (μmol/L)	CR (μmol/L)
Saline	48.69 ± 5.66	244.71 ± 22.05	33.66 ± 4.42
PAS	49.37 ± 4.53	233.42 ± 20.97	30.29 ± 3.67
Saline + HCl	46.39 ± 6.72	235.07 ± 26.35	31.96 ± 4.12

**Table 3:** Analysis of myocardium zymogram.

	LDH (U/L)	LDH1 (U/L)	CK (U/L)
Saline	1438.18 ± 73.86	86.43 ± 12.01	2697.21 ± 155.35
PAS	1410.69 ± 59.76	84.63 ± 6.37	2538.49 ± 133.18
Saline + HCl	1470.50 ± 109.24	82.19 ± 16.52	2579.21 ± 157.62

## Data Availability

No new data were created or analyzed in this study. Data sharing is not applicable to this article.

## Supplementary Materials

The following supporting information can be downloaded at: https://www.mdpi.com/article/10.3390/biomedicines10030528/s1. Plasma spectral emission intensity of DBD plasma device data in Table S1. The Metabolome analysis data was in Tables S2–S5.
